# Supplementary figures and images for: Causal associations between rheumatoid arthritis, cataract and glaucoma in European and East Asian populations: A bidirectional two-sample mendelian randomization study
Source: PLoS One. 2024 Mar 4;19(3):e0299192. doi: 10.1371/journal.pone.0299192 (PMC10911615; doi:10.1371/journal.pone.0299192)

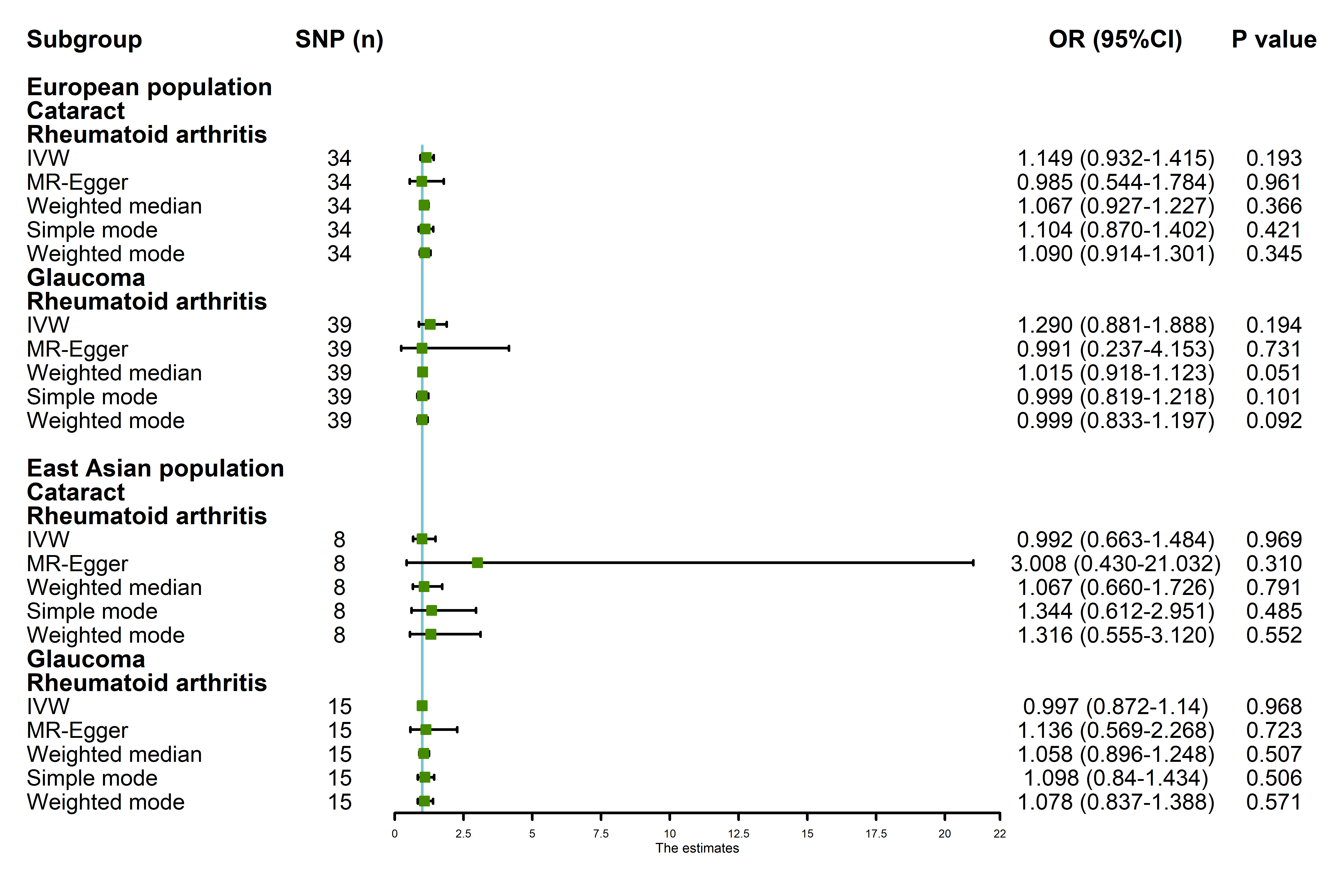

Supplement: S1 Fig — SNP (n): the number of single-nucleotide polymorphisms; RA: rheumatoid arthritis; OR: odds ratio; CI: confidence interval; IVW: inverse variance weighted. (TIF) [file pone.0299192.s001.tif]

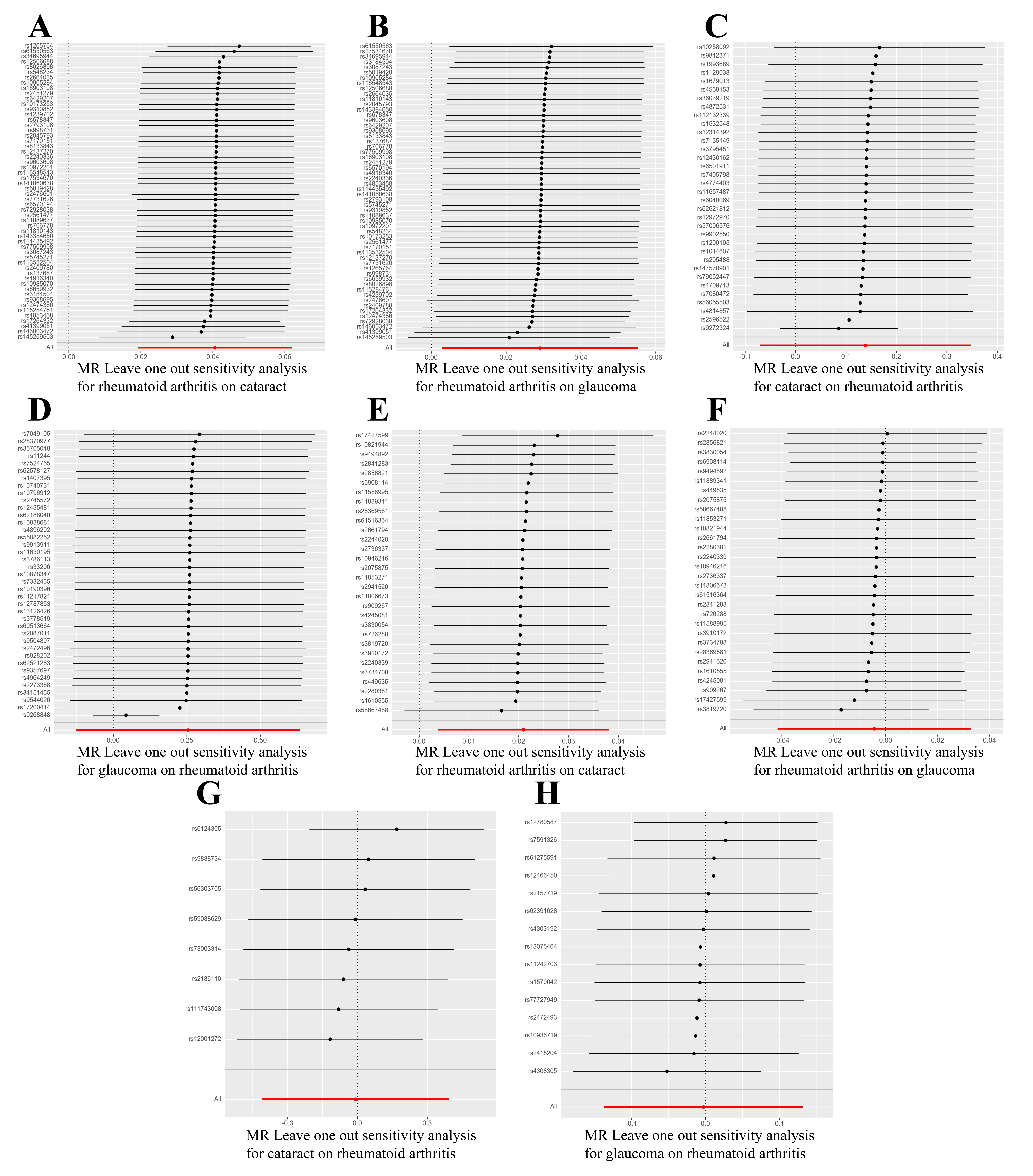

Supplement: S3 Fig — (A) Leave-one-out sensitivity test of RA on cataract in the European population; (B) Leave-one-out sensitivity test of RA on glaucoma in the European population; (C) Leave-one-out sensitivity test of cataract on RA in the European population; (D) Leave-one-out sensitivity test of glaucoma on RA in the European population; (E) Leave-one-out sensitivity test of RA on cataract in the East Asian population; (F) Leave-one-out sensitivity test of RA on glaucoma in the East Asian population; (G) Leave-one-out sensitivity test of cataract on RA in the East Asian population; (H) Leave-one-out sensitivity test of glaucoma on RA in the East Asian population. RA: rheumatoid arthritis. (TIF) [file pone.0299192.s003.tif]
